# LncRNA HABON promoted liver cancer cells survival under hypoxia by inhibiting mPTP opening

**DOI:** 10.1038/s41420-022-00917-6

**Published:** 2022-04-06

**Authors:** Lulu Wo, Xin Zhang, Chengning Ma, Cixiang Zhou, Jingchi Li, Zhexuan Hu, Xiufeng Gong, Mengna Zhan, Ming He, Qian Zhao

**Affiliations:** 1 Department of Pathophysiology, Key Laboratory of Cell Differentiation and Apoptosis of Ministry of Education, Shanghai Frontiers Science Center of Cellular Homeostasis and Human Diseases, Shanghai Jiao Tong University School of Medicine (SJTU-SM), 200025 Shanghai, China; 2 Department of Pathology, Zhongshan Hospital, Fudan University, 200031 Shanghai, China

**Keywords:** Cancer microenvironment, Cancer

## Abstract

Hypoxia is an important feature of the tumor microenvironment (TME). While targeting hypoxic TME is emerging as a potential strategy for treating solid tumors including liver cancer. Recent studies have shown that hypoxia can regulate tumor adaptation to hypoxic TME through long non-coding RNA (lncRNA). In the previous study, we identify a novel hypoxia-activated lncRNA and termed it as HABON. Here, we demonstrated that knockdown of HABON caused necroptosis of tumor tissue and inhibited the subcutaneous tumor growth of SMMC-7721 cells in nude mice. Moreover, knockdown of HABON increased RIPK1 and MLKL expression as well as their phosphorylation level in SMMC-7721 and Huh7 liver cancer cells. Meanwhile, Necrostatin-1 and GSK872 could restore cell death of liver cancer cells caused by knockdown of HABON under hypoxia. The above results suggested that HABON could inhibit hypoxia-induced necroptosis of liver cancer cells. Mechanically, knockdown of HABON in liver cancer cells aggravated mitochondrial dysfunction caused by hypoxia. Furthermore, the RNA pull-down combined with mass spectrometry analysis identified HABON can interact with mitochondria-related protein VDAC1 and the RNA immunoprecipitation (RIP) analysis proved the interaction. In addition, we proved that VDAC1 mediated the mitochondrial permeability transition pore (mPTP) opening, mitochondrial dysfunction, as well as necroptosis caused by knockdown of HABON. Overall, our work demonstrates HABON can reduce hypoxia-induced necroptosis of liver cancer cells and suggests that inhibition of HABON in the hypoxic TME is a potential therapeutic strategy for treating liver cancer.

## Introduction

Primary liver cancer is the second lethal tumor, of which more than 50% of the deaths caused by liver cancer occur in China. The misdiagnosis rate of early liver cancer is as high as 40%, and the average life expectancy of patients with liver cancer is less than 2 years. At present, surgery is still the main treatment for patients in the early and middle stages. Patients in the late stage can benefit from systemic treatment, but the effect is limited, and the 5-year survival rate is 18% [1, 2]. Hypoxia is an important environmental factor in tumors. Fifty to sixty percent of solid tumors contain hypoxic areas. According to the tumor type, the oxygen partial pressure in the tumor fluctuates from no oxygen to 60 mmHg (8% O_2_). However, most tumor cells are exposed to oxygen partial pressure from no oxygen to 7.5 mmHg (1% O_2_), which is called hypoxia [3]. Because the growth of liver cancer cells often exceeds the growth of functional blood vessels, there is often insufficient O_2_ supply in cancer tissue areas. The median oxygen partial pressure in human liver cancer tissue is 6 mmHg, while that in normal liver is 30 mmHg. Hypoxic tumors are usually more aggressive, and hypoxia limits the effectiveness of drug treatments [4, 5]. It is urgent and necessary to deeply study the molecular mechanism of hypoxia in the occurrence and development of liver cancer.

Only 2% of mammalian genomes are transcribed into protein-encoding mRNA [6], and most of the mammalian genome is transcribed into non-coding RNA [7]. LncRNAs with a length of more than 200 nucleotides are an important part of non-coding RNA. The human genome encodes more than 28,000 differently lncRNAs, but there are still many unannotated, and only a few lncRNAs have clear functions [8, 9]. In tumor tissues, hypoxia can regulate the expression of a few lncRNAs. HIF-1α can upregulate HOTAIR [10], NEAT1 [11], UCA1 [12], MALAT1 [13], H19 [14], and LincROR [15] in tumor cells by specifically binding to the HREs of their promoter [10–15]. For example, HIF-1α induces the expression of lincRNA-p21 under hypoxic conditions, and lincRNA-p21 can prevent the direct binding of HIF-1α with VHL and maintain the stability of HIF-1α, thus promote tumor survival by increasing the level of intracellular glycolysis [16].

Mitochondria are intracellular double membrane organelles, and are also the main place for aerobic respiration of eukaryotic cells. More than 90% of cellular ATP comes from mitochondria. In addition, mitochondria are also involved in the regulation of intracellular calcium homeostasis, ROS generation, cell signal transduction and cell death regulation, as well as the regulation of biosynthesis metabolism [17, 18]. The multiple functional properties of mitochondria make it an important stress sensor, which make cells adapt to the environment including hypoxic environment. Mitochondria can sense and integrate stress signals, and further regulate nuclear gene expression and intracellular protein balance [19, 20]. Recent studies have shown that lncRNA can affect mitochondrial metabolism by interacting with mitochondrial metabolism and mitochondrial translation related proteins. For example, SAMMSON is specifically expressed in melanoma, which can transport P32 protein that maintains mitochondrial homeostasis and integrity, promotes protein synthesis and significantly enhances mitochondrial activity, and then inhibits tumor cell apoptosis [21]. LncRNA such as HOTAIR, MEG2 can also affect tumor cell survival by affecting the expression of proteins related to mitochondrial apoptosis pathway [22–25].

Although a few studies have proved that hypoxia-induced lncRNA can participate in the response of tumor cells to hypoxic environment, and then affect the tumorigenesis and tumor development. However, there are few reports on the regulation of mitochondrial function by hypoxia-induced lncRNA, and their function in tumor cells under hypoxia needs to be further explored. In the previous work, we have identified a novel lncRNA which is upregulated by hypoxia, and named it HABON. Further, we prove that the upregulation of HABON under hypoxia depends on the binding of HIF-1α to HRE of its promoter, and meanwhile HABON can regulate HIF-1α protein stability and transcriptional activity [26]. In this study, we show that HABON can reduce hypoxia-induced necroptosis of liver cancer cells and reveal its mechanism is to inhibiting mPTP opening of mitochondria.

## Results

### HABON inhibited hypoxia-induced necroptosis of liver cancer cells

**Fig. 1:**
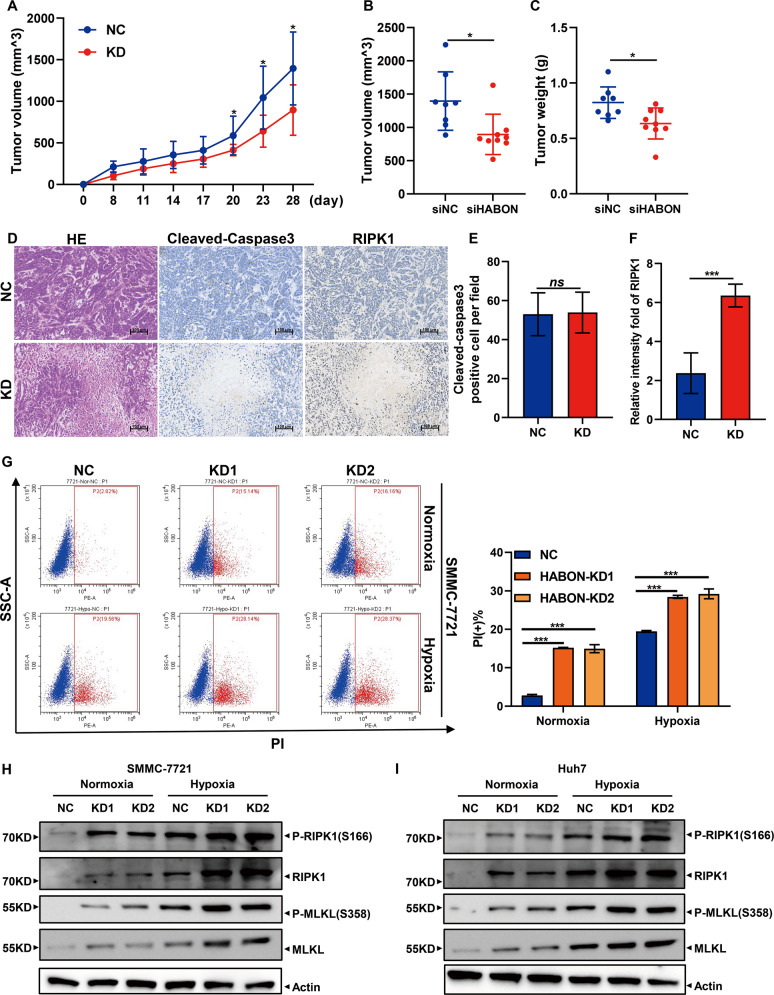
HABON inhibited hypoxia-induced necroptosis of liver cancer cells. **A**–**C** SMMC-7721 siHABON and control cells (2 × 10^6^) were injected subcutaneously into the right axilla of male nude mice for observation of tumor growth. Measure and record subcutaneous tumor size every four days. Final tumor volume and tumor weight were measured at 28 days after injection. *n* = 10 per group. **D**–**F** The representative H&E and IHC staining images of xenograft tumors. IHC staining assay was performed to detect the expression of cleaved-caspase3 and RIPK1 in the xenograft tissues. Cleaved-caspase3 and RIPK1positive cells were quantified (five fields per sample). Scale bar = 100 μm. Error bars stand for mean ± SD. Two-tailed Student’s *t*-test. *p < 0.05, **p < 0.01, ***p < 0.001. **G** Knock-down expression of HABON in SMMC-7721 cells and cell death was further detected by flow cytometry after PI staining after cultured under normoxia or hypoxia (1% O_2_) for 24 h. And the statistics of PI positive cells were shown on the right. Error bars stand for mean ± SD. Three independent experiments, two-tailed Student’s *t*-test. **H**, **I** Protein expression levels of necroptosis marker RIPK1, phospho-RIPK1 (Ser166), MLKL and phospho-MLKL (Ser358) were measured by western blot following by knockdown HABON expression in liver cancer cells cultured under normoxia or hypoxia.

To study the biological functions of HABON in liver cancer, we conducted a series of in vivo and in vitro experiments. First, we performed subcutaneous tumor formation experiments in nude mice. The negative control and HABON siRNA were transfected respectively into SMMC-7721 cells, and 48 h after transfection, the cells (2 × 10^6^) of each group were injected subcutaneously in 8-week male nude mice. The results showed that knockdown the expression of HABON can inhibit the growth of subcutaneous tumors (Fig. 1A–C). More importantly, the H&E staining showed that there was a larger necrotic area in the tumor tissue of the HABON knockdown group. Subsequently, in order to clarify the type of tumor cell death, we performed cleaved-caspase3 and RIPK1 immunohistochemistry on tumor tissues. The results showed that the expression of necroptosis marker protein RIPK1 was upregulated in the tumor tissues of the knockdown group, but there was no difference in the expression of apoptosis marker cleaved-caspase3 between the two groups (Fig. 1D–F). The above experiments suggested that knockdown of HABON could inhibit the growth of subcutaneous tumors in nude mice and cause tumor tissue necrosis.

Based on the above in vivo results, we further verified the function of HABON in liver cancer cells under hypoxia through the following in vitro experiments. After knockdown the expression of HABON in liver cancer cells SMMC-7721, Huh7, and hepG2 cells, firstly we measured the cell proliferation of each group. The results showed that knockdown of HABON could lead to a decrease of viable cell number (Supplemental Fig. 1A–I). Furthermore, we verified the effect of HABON on cell survival by PI flow cytometry analysis. The results showed that knockdown of HABON promoted hypoxia-induced cell death of SMMC-7721 cells (Fig. 1G) as well as Huh7 and hepG2 cells (Supplemental Fig. 1J, K). Meanwhile, we detected the expression of necroptosis markers RIPK1, phospho-RIPK1 (Ser166), and MLKL and phospho-MLKL (Ser358). The Western Blot results showed that knockdown of HABON further increased both the expression and phosphorylation of RIPK1 and MLKL induced by hypoxia (Fig. 1H–I). To further investigate the function of HABON in regulating cell death under hypoxia, we overexpressed HABON in SMMC-7721, Huh7, and hepG2 cells and performed the related experiments. The results showed that overexpression of HABON could inhibit both the decrease in the number of viable cells and increase cell death caused by hypoxia in these three liver cancer cell lines (Supplemental Fig. 2A–F). These results suggested HABON could inhibit hypoxia-induced necroptosis of liver cancer cells.

### Necroptosis inhibitors rescued cell death caused by knockdown of HABON under hypoxia

**Fig. 2:**
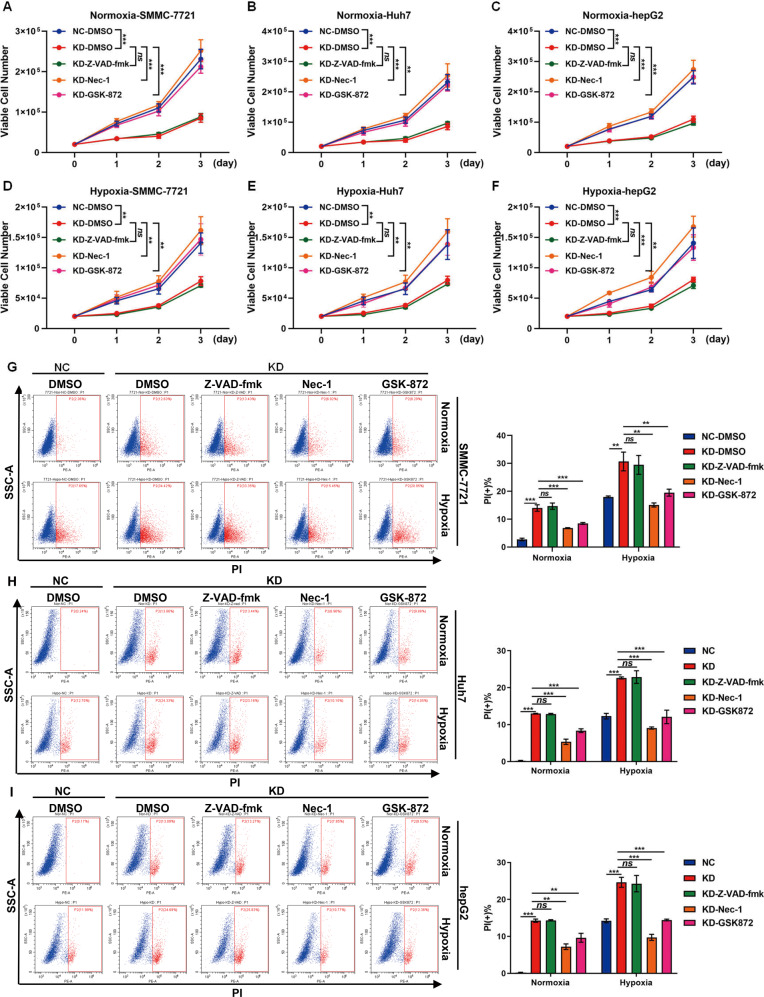
Necroptosis inhibitors can rescue cell death caused by knockdown of HABON under hypoxia. **A**–**F** The expression of HABON was knockdown in SMMC-7721, Huh7 and hepG2 cells. The viable cells were counted after cultured under normoxia or hypoxia (1% O_2_) and treated with apoptosis inhibitor (20 μM Z-VAD-fmk) or necroptosis inhibitor (20 μM Nec-1, 5 μM GSK872) for different time. Three independent experiments, two-tailed Student’s *t*-test. **G**–**I** Flow cytometry was used to detect cell death after PI staining following treated with apoptosis or necroptosis inhibitor for 24 h. And the statistics of PI positive cells were shown. Error bars stand for mean ± SD. Three independent experiments, two-tailed Student’s *t*-test. *p < 0.05, **p < 0.01, ***p < 0.001.

To further confirm that knockdown of HABON caused necroptosis of liver cancer cells, we used the following inhibitors to treat SMMC-7721, Huh7, and hepG2 cells respectively and perform viable cell count as well as PI flow cytometry analysis. As well known, Necrostatin-1 (Nec-1) is a RIPK1 inhibitor, GSK872 is a RIPK3 inhibitor, and Z-VAD-fmk is a pan-Caspase inhibitor. The results of viable cell count showed that both Nec-1 and GSK872 treatment can restore the decrease of viable cell number caused by knockdown of HABON under hypoxia, while the apoptosis inhibitor Z-VAD-fmk has no effect on this (Fig. 2A–F). The results of PI flow cytometry analysis also indicated that both Nec-1 and GSK872 can restore cell death caused by knockdown of HABON, while the apoptosis inhibitor Z-VAD-fmk has no effect on this (Fig. 2G–I). Taken together, the above results further confirmed that HABON inhibited hypoxia-induced necroptosis of liver cancer cells.

### HABON inhibited hypoxia-induced damage of mitochondrial functions

To analyze why HABON could inhibit hypoxia-induced necroptosis of liver cancer cells, we firstly evaluated the mitochondrial function. As well known, hypoxia can cause damage to mitochondrial function, which leads to decreased ATP content, increased ROS, decreased mitochondrial transmembrane potential, increased oxidative damage, and accumulation of mitochondrial DNA mutations [27].

**Fig. 3:**
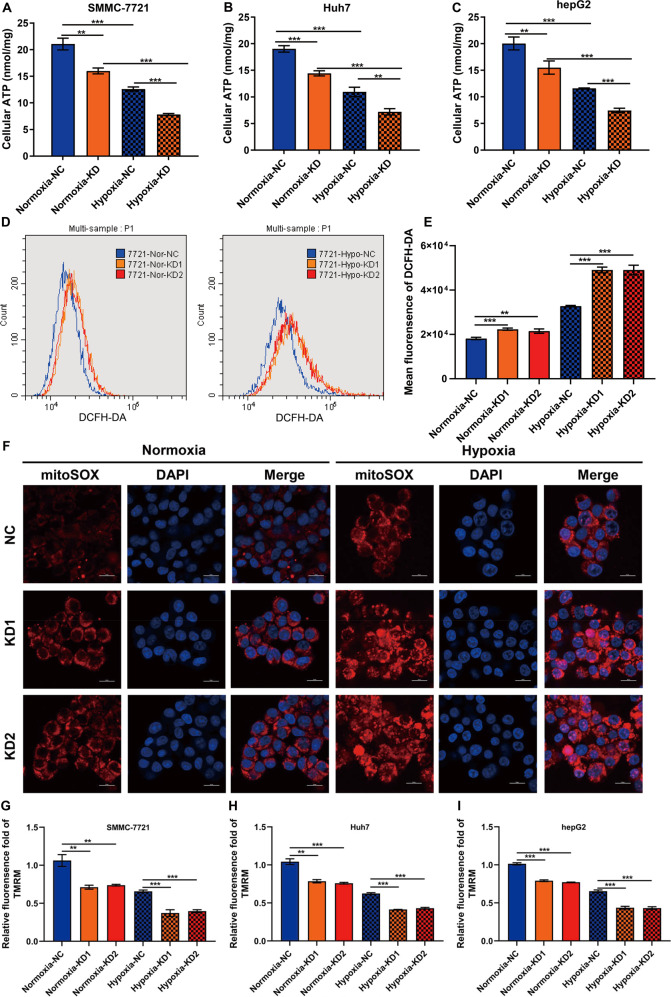
HABON inhibited hypoxia-induced damage of mitochondrial function. **A**–**C** siHABON or siNC were transfected into SMMC-7721, Huh7, and hepG2 cells. The cells were cultured under normoxia or hypoxia for 24 h and the cellular ATP content was measured. **D**, **E** Determination of cellular ROS content in each group of SMMC-771 cells by DCFH-DA flow cytometry. **F** Perform MitoSox staining and mitochondrial ROS level in each group of hepG2 cells was visualized by confocal microscopy. **G**–**I** Mitochondrial membrane potential in each group of SMMC-771 cells was measured by TMRM flow cytometry and relative fluorescence intensity was calculated. Error bars stand for mean ± SD. Three independent experiments, two-tailed Student’s *t*-test. *p < 0.05, **p < 0.01, ***p < 0.001.

Based on this, we studied whether HABON is involved in the hypoxia-induced damage of mitochondrial function. We first detected the ATP content. After transfection the negative control or HABON siRNA into the SMMC-7721, Huh7, and hepG2 cells respectively, we treated the samples of each group under normoxia or hypoxia for 24 h. As expected, the cellular ATP contents were reduced in hypoxia-treated groups compared to the normoxia groups, while knockdown of HABON led to a further decrease of cellular ATP contents (Fig. 3A–C). Meanwhile, mitochondrial ATP content detection also showed that knockdown of HABON caused a further decrease in mitochondrial ATP contents caused by hypoxia (Supplemental Fig. 3A–F). In addition, DCFH-DA flow cytometry assay and mitoSOX were used to detect intracellular and mitochondrial ROS in liver cancer cells, respectively. The results showed that knockdown of HABON led to a further increase in intracellular and mitochondrial ROS caused by hypoxia (Fig. 3D–F and Supplemental Fig. 3G, H). We performed TMRM flow cytometry assay to detect the mitochondrial transmembrane potential of each group of samples and quantified their fluorescence intensity. The results showed that knockdown of HABON aggravated the decrease of mitochondrial transmembrane potential caused by hypoxia in SMMC-7721, Huh7, and hepG2 cells (Fig. 3G–I). Collectively, the above results indicated that HABON could inhibit the damage of mitochondrial functions caused by hypoxia in liver cancer cells.

### HABON interacted with VDAC1 on mitochondria

**Fig. 4:**
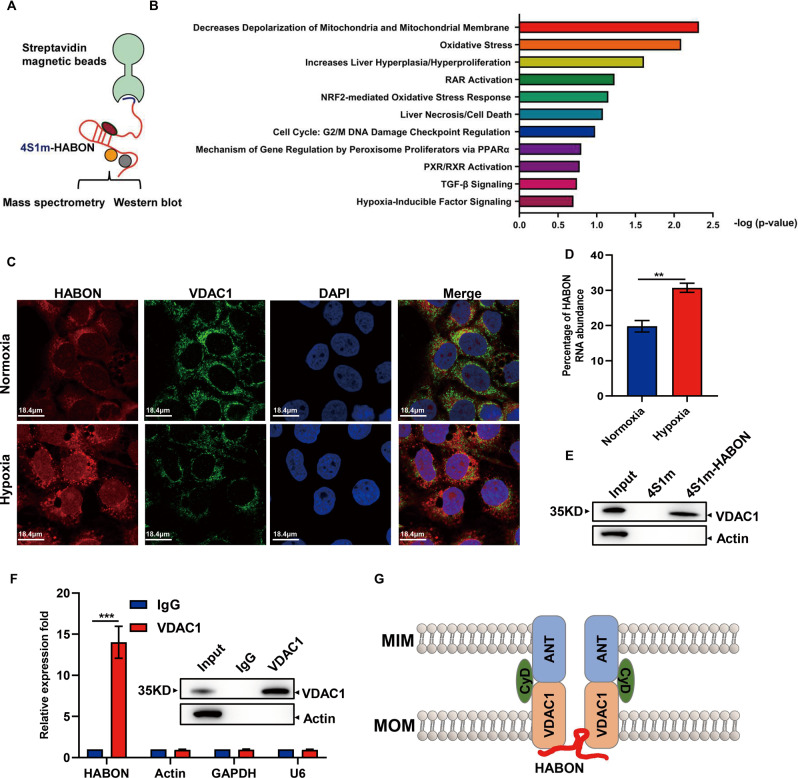
HABON interacted with VDAC1 on mitochondria. **A** 4XS1m-HABON or 4XS1m was expressed in Huh7 cells under hypoxia treatment for 24 h. Use streptavidin magnetic beads for immunoprecipitation experiments. Mass spectrometry were performed after protein gel electrophoresis and the binding protein were further verified by western blot. **B** Use the IPA (Ingenuity Pathway Analysis) bioinformatics website to perform functional enrichment analysis on the protein binding with HABON. **C** Huh7 cells were treated with normoxia and hypoxia for 24 h. After the samples were fixed, HABON RNA FISH and VDAC1 immunofluorescence experiments were performed, and they were observed under a confocal microscope. The scale bar is 18.4 μm. **D** Isolation of mitochondria in Huh7 cells cultured under normoxia and hypoxia for 24 h. qRT-PCR was used to detect the expression of HABON in the mitochondrial components. **E** 4XS1m-HABON or 4XS1m was expressed in Huh7 cells with hypoxia treatment for 24 h. Use streptavidin magnetic beads for immunoprecipitation experiments. Western blot verified the combination of HABON with VDAC1. **F** Huh7 cells were cultured under hypoxia for 24 h an RIP experiment was performed with anti-VDAC1 antibody or IgG. qRT-PCR detected the enrichment of VDAC1 on HABON and negative controls Actin, GAPDH and U6 in RNA samples. **G** Schematic of the colocalization of HABON with VDAC1 on outer mitochondrial membrane. Error bars stand for mean ± SD. Three independent experiments, two-tailed Student’s *t*-test. *p* < 0.05, **p < 0.01, ***p < 0.001.

To further investigate the molecular mechanism of HABON in the regulation of mitochondrial function as well as necroptosis of liver cancer cells. We performed RNA pull-down of HABON, followed by mass spectrometry (MS) assay between Huh7 cells with hypoxia treatment to identify the proteins interacting with HABON. We analyzed the mass spectrometry results of RNA pull-down by IPA. The IPA analysis indicated that the proteins enriched by HABON are involved in regulating mitochondrial function and cell death related pathways, such as mitochondrial depolarization, oxidative stress, cell proliferation, and necrosis (Fig. 4A, B). In view of the above analysis, it is suggested that the proteins enriched by HABON are mainly involved in the regulation of mitochondrial function, we firstly analyzed the intracellular localization of HABON. We used mitochondrial outer membrane protein VDAC1 as a mitochondrial marker, performed VDAC1 immunofluorescence and HABON in situ hybridization. The results showed that HABON was mainly localized in the cytoplasm under normoxia in Huh7 cells while its expression and colocalization with mitochondria was increased under hypoxia (Fig. 4C). In addition, in order to verify the above results, we isolated mitochondria and detected the distribution of HABON in mitochondria. The results also suggested that hypoxia treatment increased the localization of HABON on mitochondria in Huh7 cells (Fig. 4D). According to the functional enrichment analysis of HABON interacting proteins and the localization analysis of HABON, we further screened VDAC1 as a candidate for HABON interacting protein. VDAC1, the most abundant protein in the outer membrane of mitochondria, is the gatekeeper of metabolites, nucleotides, and ion channels. At the same time, it plays a vital role in cell death [28]. To verify the interaction of HABON and VDAC1, firstly, we conducted the RNA pull-down and the results suggested that HABON could enrich VDAC1 (Fig. 4E). Consistently, The HABON-VDAC1 complex was further validated by RNA immunoprecipitation (RIP) followed by q-RT-PCR analysis and the results showed that VDAC1 could enrich HABON under hypoxia, but not the negative controls (Fig. 4F). In conclusion, our results demonstrated that HABON could localized on mitochondria and bind with VDAC1 under hypoxia treatment (Fig. 4G).

### Damage of mitochondrial function resulted from knockdown of HABON was mediated by mPTP opening

**Fig. 5:**
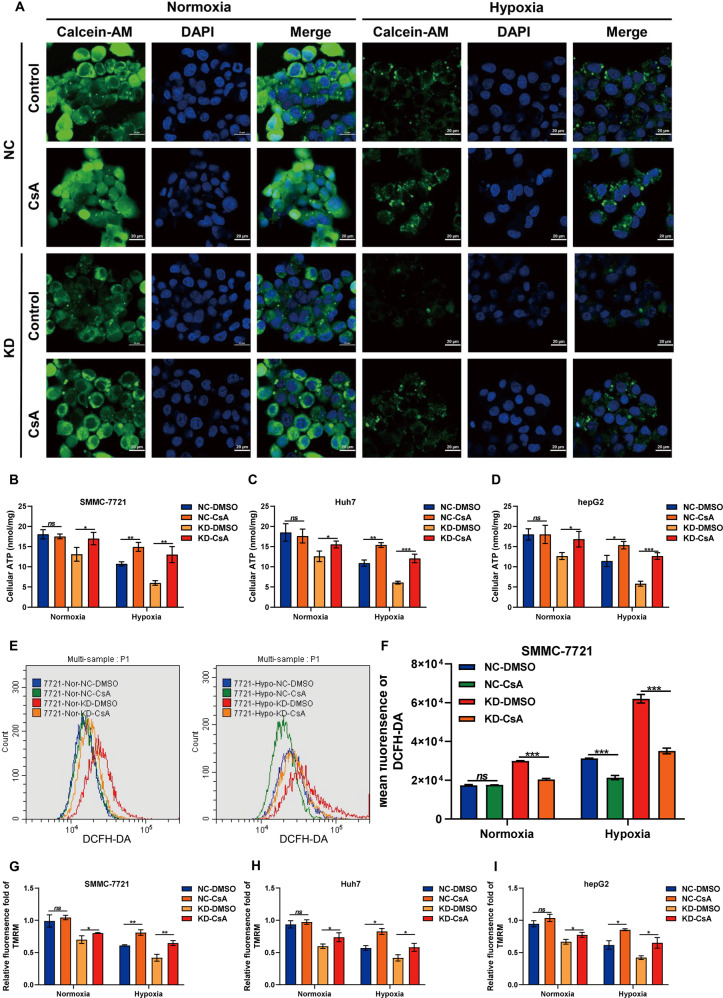
Damage of mitochondrial function resulted from knockdown of HABON under hypoxia was mediated by mPTP opening. **A** Transfect siHABON or its control into liver cancer cells, and cells were cultured under normoxia or hypoxia with or without CsA (5 μM) for 24 h. Perform mPTP assay in each group of hepG2 cells and the opening of mPTP was visualized and measured by confocal microscopy. **B**–**D** Measure the cellular ATP content. Three independent experiments, two-tailed Student’s *t*-test. **E**, **F** Quantification of ROS content in each group of SMMC-771 cells by DCFH-DA flow cytometry. And the statistics of mean fluorescence were shown. **G**–**I** Mitochondrial membrane potential in each group of samples were measured by TMRM flow cytometry and relative fluorescence intensity was calculated. Error bars stand for mean ± SD. Three independent experiments, two-tailed Student’s *t*-test. *p < 0.05, **p < 0.01, ***p < 0.001.

VDAC1 has been reported to play important roles in regulating mitochondrial function as well as cell death by regulating opening of mitochondrial permeability transition pore (mPTP) [29, 30]. To further verify whether the damage of mitochondrial function caused by knockdown of HABON under hypoxia is mediated by VDAC1, we knockdown HABON in liver cancer cells, and treated samples with mPTP inhibitor Cyclosporin A (CsA). Calcein AM was used to indicate the statues of mPTP and the results showed that CsA could inhibit the opening of mPTP caused by knockdown of HABON under hypoxia (Fig. 5A and Supplementary Fig. 4A). The results of cellular ATP detection showed that CsA could inhibit the reduction of cellular ATP content induced by knockdown of HABON under hypoxia (Fig. 5B–D). Meanwhile, mitochondrial ATP assay results also showed that CsA could inhibit the reduction of mitochondrial ATP content induced by knockdown of HABON (Supplementary Fig. 4B–D). In addition, the DCFH-DA flow cytometry assay and mitoSOX indicated that CsA impaired the increase of intracellular and mitochondrial ROS caused by knockdown of HABON under hypoxia (Fig. 5E, F. Supplementary Figs. 4E–H and 5). Moreover, the TMRM flow cytometry assay showed that CsA could inhibit the reduction of mitochondrial transmembrane potential induced by knockdown of HABON under hypoxia (Fig. 5G–I). Taken together, the above results suggested that VDAC1 mediated the damage of mitochondrial function induced by knockdown of HABON in liver cancer cells under hypoxia treatment.

### Necroptosis caused by knockdown of HABON under hypoxia was mediated by mPTP opening

**Fig. 6:**
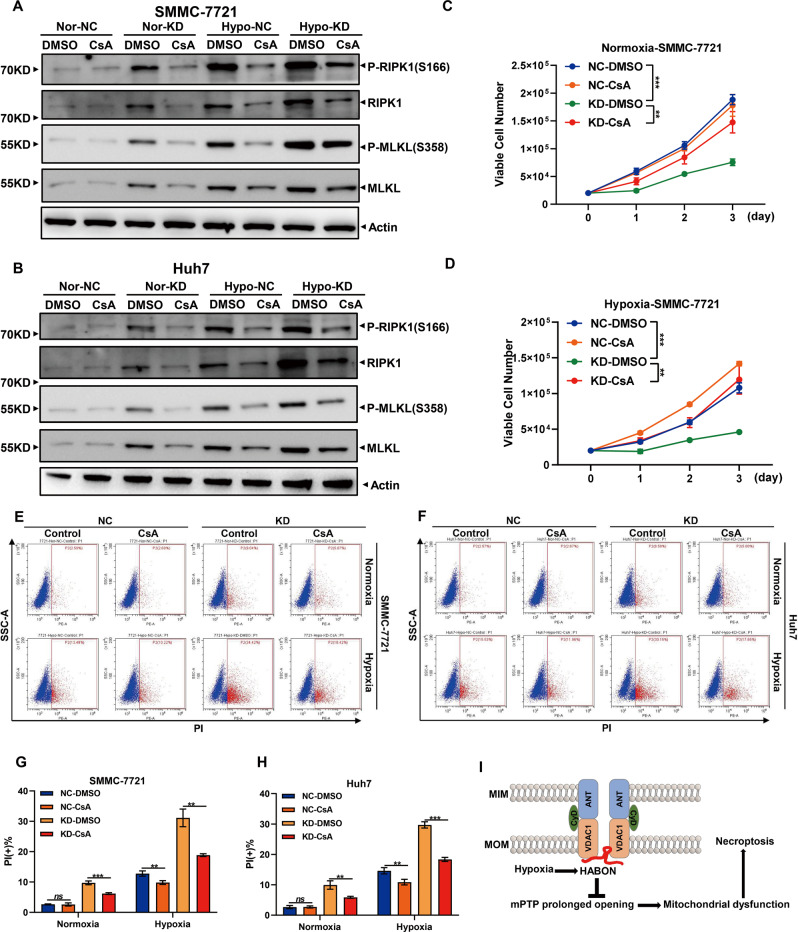
Necroptosis caused from knockdown of HABON under hypoxia was mediated by mPTP opening. **A**, **B** Expression levels of necroptosis marker RIPK1, phospho-RIPK1 (Ser166), MLKL, and phospho-MLKL (Ser358) were measured by western blot following by knockdown HABON expression in liver cancer cells with or without hypoxia treatment. **C**, **D** The expression of HABON was knock-down in SMMC-7721 cells. The viable cells were measured after cultured under normoxia or hypoxia (1% O_2_) and treated with mPTP inhibitor (5 μM CsA) for different time. Three independent experiments, two-tailed Student’s *t*-test. **E**, **F** Flow cytometry was used to detect cell death of SMMC-771 and Huh7 cells after PI staining following treated with mPTP inhibitor (5 μM CsA) for 24 h. The statistics of PI positive cells were shown in **G**, **H**. Error bars stand for mean ± SD. Three independent experiments, two-tailed Student’s *t*-test. *p < 0.05, **p < 0.01, ***p < 0.001. **I** The mechanism schematic of HABON involved in the regulation of necroptosis induced by hypoxia.

Meanwhile, we verified the role of mPTP opening in necroptosis of liver cancer cells caused by knockdown of HABON. Western blot showed that CsA could restore the increased expression and phosphorylation of RIPK1 and MLKL caused by knockdown of HABON under hypoxia (Fig. 6A, B). The results of live cell count experiments showed that CsA could inhibit the decrease of viable cell number caused by knockdown of HABON under hypoxia (Fig. 6C, D and Supplementary Fig. 6A–D). Moreover, PI flow cytometry assay demonstrated that CsA could inhibit the necroptosis of liver cancer cells caused by knockdown of HABON under hypoxia (Fig. 6E–H and Supplementary Fig. 6E). In summary, the above results proved that the opening of mPTP mediated the function of HABON to inhibit the necroptosis of liver cancer cells under hypoxia and the mechanism schematic was shown in Fig. 6I.

## Discussion

Hypoxia is one of the important microenvironment factors in many solid tumors, HIF-1 α is the main regulatory molecule in tumor under acute hypoxia (generally within 24 h). On one hand, HIF-1α can alleviate hypoxia by promoting angiogenesis, on the other hand, HIF-1α can also lead to tumor necrosis. Chronic hypoxia of tumor is mainly mediated by HIF-2α and promote tumor proliferation and progression. The effects of hypoxia on tumor survival are diverse and the mechanism is complicated, which needs to be further studied. The results of subcutaneous xenograft suggest that there are two forms of cell death: necroptosis and apoptosis. The expression of RIPK1 mediating necroptosis in tumor tissue of HABON knockdown group was significantly upregulated, but there was no difference in apoptosis between the two groups. As well known, necroptosis is mediated by the binding of TNFα with its receptor TNFR1 and a series of downstream complexes. Morphologically, it is characterized by swelling of cells and organelles represented by mitochondria [31]. Different complexes can lead to cell survival, apoptosis, and necroptosis [32, 33]. After TNFα binding to TNFR1, the cytoplasmic end of TNFR1 recruits RIPK1, TRADD, CIAP, TRAF2, and LUBAC to form complex I and activate NF-κB to promote cell survival [34, 35]. If RIPK1 in complex I is de-ubiquitinated and dissociated from the cell membrane, it forms complex IIA with pro-caspase-8, TRADD and FADD and causes apoptosis [36]. If RIPK1 is phosphorylated, it forms complex IIB with RIPK3, pro-caspase-8 and TRADD, which can also cause apoptosis. If RIPK1 kinase activity is high enough or caspase-8 activity is reduced or deleted during this process, RIPK3 in complex IIb is phosphorylated and leads to necroptosis through recruitment and phosphorylation of MLKL [33, 37, 38]. These suggest that there is a complex regulation between cell survival, apoptosis, and necroptosis, thus the role and mechanism of HABON in this process is needed to be further studied.

In addition, our results showed that the co-localization of HABON with mitochondria is increased under hypoxia treatment. Meanwhile HABON could interact with mitochondrial outer membrane protein VDAC1, and promote the survival of liver cancer cells by inhibiting the opening of mPTP. VDAC1 located in the outer membrane of mitochondria and is involved in the regulation of mitochondrial material transport, therefore plays an important role in the regulation of membrane permeability and cell death. As mentioned earlier, the opening of mPTP can lead to the release of cytochrome c and further lead to apoptosis [39]. However, it has also been reported that mPTP complex also leads to autophagic cell death by activating mitophagy [40]. In addition, VDAC1 has been proved to be a substrate of PINK1-PARKIN pathway, which mediates mitophagy. It mediates mitophagy by binding p62, OPTN, and LC3-II [39–42]. Therefore, whether HABON is involved in regulating other functions of VDAC1 remains to be further explored.

In conclusion, the upregulated lncRNA HABON under hypoxia inhibits hypoxia-induced necroptosis of liver cancer cells by interacting with VDAC1 and regulating the opening of mPTP. These studies have important implications for understanding the role of lncRNA in hypoxic response of liver cancer cells and provide new clues to the treatment of liver cancer.

## Materials and methods

### Cell lines and cell culture

Liver cancer cell lines SMMC-7721 and Huh7 are from Shanghai cell bank, Chinese Academy of Sciences. hepG2 is provided by Xuemei tong (SJTU-SM). The above cells were cultured in a 5% CO2, 37 °C constant temperature cell incubator using DMEM high-sugar medium containing 10% FBS. When performing hypoxia treatment in the experiment, the cells were placed in a cell incubator with 1% O_2_, 5% CO_2_, and 37 °C. When performing CsA (Selleck, 290193) treatment, the final concentration is 20 μM.

### Total RNA extraction and quantitative real-time PCR

According to the manual instructions, extract total RNA from cell lines using Trizol reagent (Invitrogen,15596018). cDNA was obtained through the AMV reverse transcription system (Takara,2621). Quantitative real-time PCR was performed using SYBR green PCR premix reagent (ABI,4472908) and specific primers of shown in Table 1. The data analysis of expression in cell lines was normalized by t β-Actin, and evaluated using 2^−∆∆Ct^ method.

### Western blot

Antibodies used in this paper: RIPK1(Proteintech,17519-1-AP), P-RIPK1(S166) (Proteintech,28252-1-AP), MLKL (CST,14993), P-MLKL (S358) (CST,91689), VDAC1 (Proteintech,55259-1-AP), β-Actin-HRP (MBL, PM053-7). The experimental protocol of sample extraction and electrophoresis was described elsewhere [26].

### Subcutaneous tumor formation

Twenty 6–8-week-old male nude mice used in this experiment were purchased from Shanghai SLAC Laboratory Animal and were adaptively housed in the Department of Animal Science, Shanghai Jiaotong University School of Medicine, and related experiments were carried out after 2 weeks. 2 × 10^6^ cells were injected into the axilla of nude mice, which were grouped randomly (*n* = 10). The size of the subcutaneous tumor was measured with a vernier caliper every 4 days, and the volume of the tumor was calculated according to the following formula: volume = length * width^2^/2. The experiment was terminated 4 weeks after the inoculation, and the subcutaneous tumor tissue was collected and weighed. All animal experiments were approved by the Animal Care and Use Committee of Shanghai Jiao Tong University School of Medicine and performed in accordance with relevant guidelines.

### H&E staining and immunohistochemistry

Tumor tissue were embedded in paraffin, and then were de-paraffinized and stained with Hematoxylin and Eosin, cleaved-Caspase3 antibody (CST, 9661) and RIPK1 antibody. For immunohistochemistry, the slides were visualized by standard avidin-biotinylated peroxidase complex method and positive cells were evaluated blindly by Image J.

### Cell count

2 × 10^4^ cells after transfection for 48 h were seeded into 24-well plate and then cultured under normoxia or hypoxia for 3 days. And count viable cell number of each group every 24 h with trypan blue staining.

### Flow cytometry assay

Seed cells in 12-well culture plate. Wash cells with cold PBS after respective treatment. Collect cells into 1.5 mL tubes and stain samples according to PI (BD, 51-66211E), DCFH-DA (Yeasen, 50101ES01) and TMRM (Invitrogen, I34361) protocol. Analyze samples by Beckman Coulter (CytoFlex S).

### Mitosox and mPTP assay

Cells were seeded in glass bottom dish. After cultured under different condition, wash samples with cold PBS and perform experiments according to the Mitosox (Yeasen, 40778ES50) and mPTP assay (Beyotime, C2009S) kit instructions. Cells were observed under Nikon T1 microscope.

### Mitochondria separation

At least 5 × 10^6^ cells were needed for this experiment. Collect cells into 1.5 mL tubes and wash with cold PBS. After 250 μL separation solution (Beyotime, C3601) is added, keep samples on ice for 10–15 min, and stop homogenizing when the positive rate of trypan blue staining is greater than 50%. Centrifuge at 4 °C, 600×*g* for 10 min and take the supernatant into a new tube. Centrifuge at 4 °C, 11,000×*g* for 10 min and the precipitate is mitochondria.

### ATP test

Cells was seeded in 6-well culture plate with respective treatment. Wash cells with cold PBS and add 300 μL lysis buffer. The lysate were collected and centrifuged at 12,000×*g* for 10 min at 4 °C. Transfer the supernatant to 1.5 mL tubes for ATP test with the ATP detection kit (Beyotime, S0026).

### RNA pull-down

Samples were collected and washed with cold PBS and 1 mL Buffer A (150 mM KCl, 25 mM Tris 7.4, 5 mM EDTA, 0.5% NP40, 0.5 M DTT 1 μL) with Protease Inhibitor Cocktail (Millipore, 539134) 10 μL, RNase Inhibitor 10 μL (TAKARA, 2313B) added. After centrifuging at 2000×*g*, 4 °C for 5 min, the lysate supernatant was incubated with Streptavidin Magnetic Beads (NEB, S1420S) at 4 °C overnight, and then washed with cold Buffer A for six times. Finally, add 2xSDS electrophoresis sample buffer to the sample. After denaturation, the sample were centrifuged at 5000×*g* for 5 min, and the supernatant was used for Western Blot or mass spectrometry analysis (Platform of Mass Spectrometry, Shanghai Jiaotong University).

### RNA immunoprecipitation

In brief, 10^7^ cells were collected and lysed in RIP lysis buffer with 1 mM DTT, PMSF, cocktail as well as RNase Inhibitor. Then whole cell lysates were incubated with RIP buffer containing Protein A/G-agarose (Santa Cruz, sc-2003) conjugated with VDAC1 antibody or negative control normal rabbit IgG (Millipore, AQ132P). Incubated with Proteinase K, the RNA was isolated using TRIzol reagent and subjected to real-time PCR using specific primers.

### RNA Fluorescent in situ hybridization and immunofluroscence staining

About 2 × 10^5^ Huh7 cells was seeded on cover slides in 24-well culture plate with or without hypoxia treatment. Then we performed the procedures according to the manufacturer’s protocol of Fluorescent In Situ Hybridization Kit (Ribo, R11060.1). For the staining of VDAC1, primary antibody was used at the dilution rate of 1:100 in 5% BSA and incubated at 4 °C overnight. A total volume of 200 μL containing 2 μL FITC-conjugated Anti-Rabbit IgG (Invitrogen, A16024) was used per well and incubated 1 h at room temperature. Slides were observed under Leica (TCS SP8 STED 3×) microscope.

### Informatics tools and statistics analysis

IPA (http://www.ingenuity.com/products/ipa#/?tab=features) was used for analysis of mass spectrometry results. Data that are normally distributed are shown as the mean ± SD. Use GraphPad Prism to process the data and assess differences (Student’s *t*-test) between experimental groups.

## Supplementary information

Supplmental legend

Supplemntal figure 1

Supplemntal figure 2

Supplemntal figure 3

Supplemntal figure 4

Supplemntal figure 5

Supplemntal figure 6

uncropped western blots

SUPPLEMENTAL Table 1

## Data Availability

All data that support the findings of this study are available from the corresponding author upon reasonable request.
