# Supplementary material for: LncRNA HABON promoted liver cancer cells survival under hypoxia by inhibiting mPTP opening
Source: Cell Death Discov. 2022 Apr 6;8:171. doi: 10.1038/s41420-022-00917-6 (PMC8986810; doi:10.1038/s41420-022-00917-6)
Supplement: Supplementary file 1 — Supplmental legend [file 41420_2022_917_MOESM1_ESM.docx]

# Supplemental Figure 1. Knockdown of HABON in SMMC-7721, Huh7 and

**hepG2 cells promoted hypoxia-induced cell death.** (A-I) Real-time PCR were performed to analyze the knock-down efficiency of HABON in SMMC-7721, Huh7 and hepG2 cells. The viable cells following HABON knockdown were counted after cultured under normoxia or hypoxia (1% O_2_) for different time. Three independent experiments, two-tailed Student’s t-test. (J-K) Flow cytometry assay were used to detect cell death of Huh7 and hepG2 cells. And the statistics of PI positive cells were shown on the right. Error bars stand for mean ± SD. Three independent experiments, two-tailed Student’s t-test. **p*<0.05, ***p* < 0.01, ****p*<0.001.

# Supplemental Figure 2. Overexpression of HABON in SMMC-7721, Huh7 and hepG2 cells inhibited hypoxia-induced cell death. (A-C) The viable cells of SMMC-7721, Huh7 and hepG2 cells were counted after HABON overexpression and cultured under normoxia or hypoxia (1% O_2_) for different time. Three independent experiments, two-tailed Student’s t-test. (D-F) Flow cytometry assay were used to detect cell death after HABON overexpression. And the statistics of PI positive cells were shown on the right. Error bars stand for mean ± SD. Three independent experiments, two-tailed Student’s t-test. **p* <0.05, ***p* < 0.01, ****p*<0.001.

**Supplemental Figure 3. Knockdown of HABON in SMMC-7721, Huh7 and hepG2 liver cancer cells promoted mitochondrial dysfunction caused by hypoxia.** (A-C) siHABON or siNC were transfected into SMMC-7721, Huh7 and hepG2 cells, and the cells were cultured under normoxia or hypoxia for 24 hours. Mitochondria of liver cancer cells cultured under normoxia or hypoxia were separated, and Western blot was used to detect the expression of Actin and TOM20 in whole cell lysis (WCL), cytosol without mitochondria (Cyto) and mitochondrial (Mito) samples. Among them, Actin is a cytoplasmic marker protein, and TOM20 is a mitochondrial marker protein. (D-F) Measure the mitochondrial. ATP content.Three independent experiments, two-tailed Student’s t-test. (G-H) Determination of ROS content in each group of Huh7 and hepG2 cells by DCFH-DA flow cytometry assay. And the statistics of mean fluorescence were shown on the right. Error bars stand for mean ± SD. Three independent experiments, two-tailed Student’s t-test. **p* <0.05, ***p* < 0.01, ****p*<0.001.

**Supplemental Figure 4. The mPTP inhibitor CsA rescued HABON knockdown-induced mitochondrial dysfunction of liver cancer cells under hypoxia.** (A) Transfect siHABON or its control into liver cancer cells, and cells were cultured under normoxia or hypoxia with or without CsA (5μM) for 24 hours. The opening of mPTP in each group of Huh7 cells was visualized and measured by confocal microscopy. (B-D) Separate mitochondria in each group of samples, and detect the mitochondrial ATP content. Three independent experiments, two-tailed Student’s t-test. (E-H) Quantification of ROS content of Huh7 and hepG2 cells by DCFH-DA flow cytometry assay. And the statistics of mean fluorescence were shown. Error bars stand for mean ± SD. Three independent experiments, two-tailed Student’s t-test. **p* <0.05, ***p* < 0.01, ****p*<0.001.

**Supplemental Figure 5. The mPTP inhibitor CsA impaired the increase of mitochondrial ROS induced by HABON knockdown**. Transfect siHABON or its control into liver cancer cells, and cells were cultured under normoxia or hypoxia with or without CsA (5μM) for 24 hours. Perform MitoSox staining and mitochondrial ROS level in each group of Huh7 (A) and hepG2 (B) cells was visualized by confocal microscopy.

# Supplemental figure 6. The mPTP inhibitor CsA rescued HABON knockdown-induced necroptosis of liver cancer cells under hypoxia. (A-D) The expression of HABON was knock-down in Huh7 and hepG2 cells. The viable cells under normoxia or hypoxia (1% O_2_) were quantified after treated with CsA (5μM) for different time. Three independent experiments, two-tailed Student’s t-test. (E) Flow cytometry assay were performed to detect cell death of hepG2 cells after treated with CsA for 24 hours. And the statistics of PI positive cells were shown. Error bars stand for mean ± SD. Three independent experiments, two-tailed Student’s t-test. **p* <0.05, ***p* < 0.01, ****p*<0.001.
