# Supplementary figures and images for: LncRNA HABON promoted liver cancer cells survival under hypoxia by inhibiting mPTP opening
Source: Cell Death Discov. 2022 Apr 6;8:171. doi: 10.1038/s41420-022-00917-6 (PMC8986810; doi:10.1038/s41420-022-00917-6)

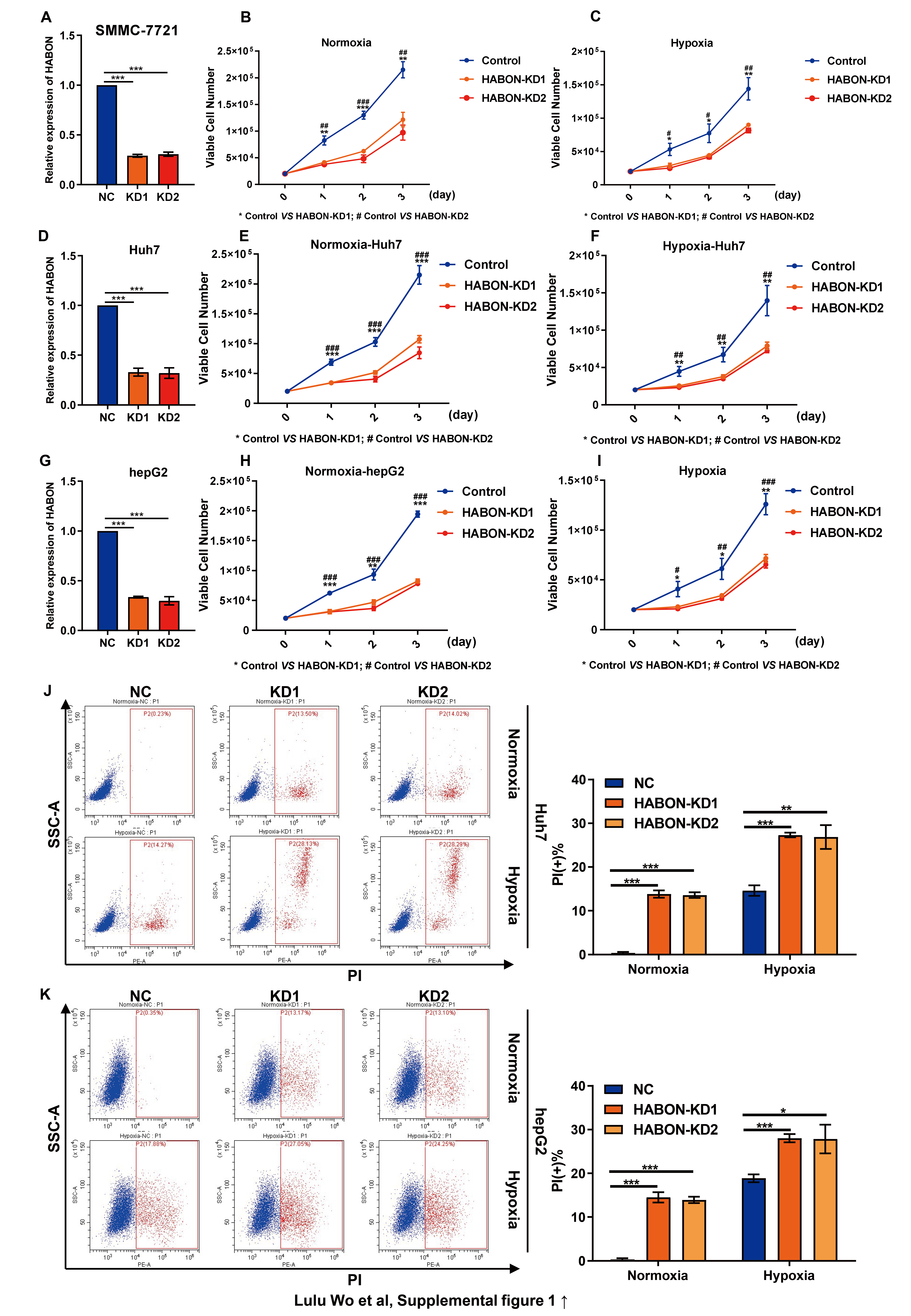

Supplement: Supplementary file 2 — Supplemntal figure 1 [file 41420_2022_917_MOESM2_ESM.png]

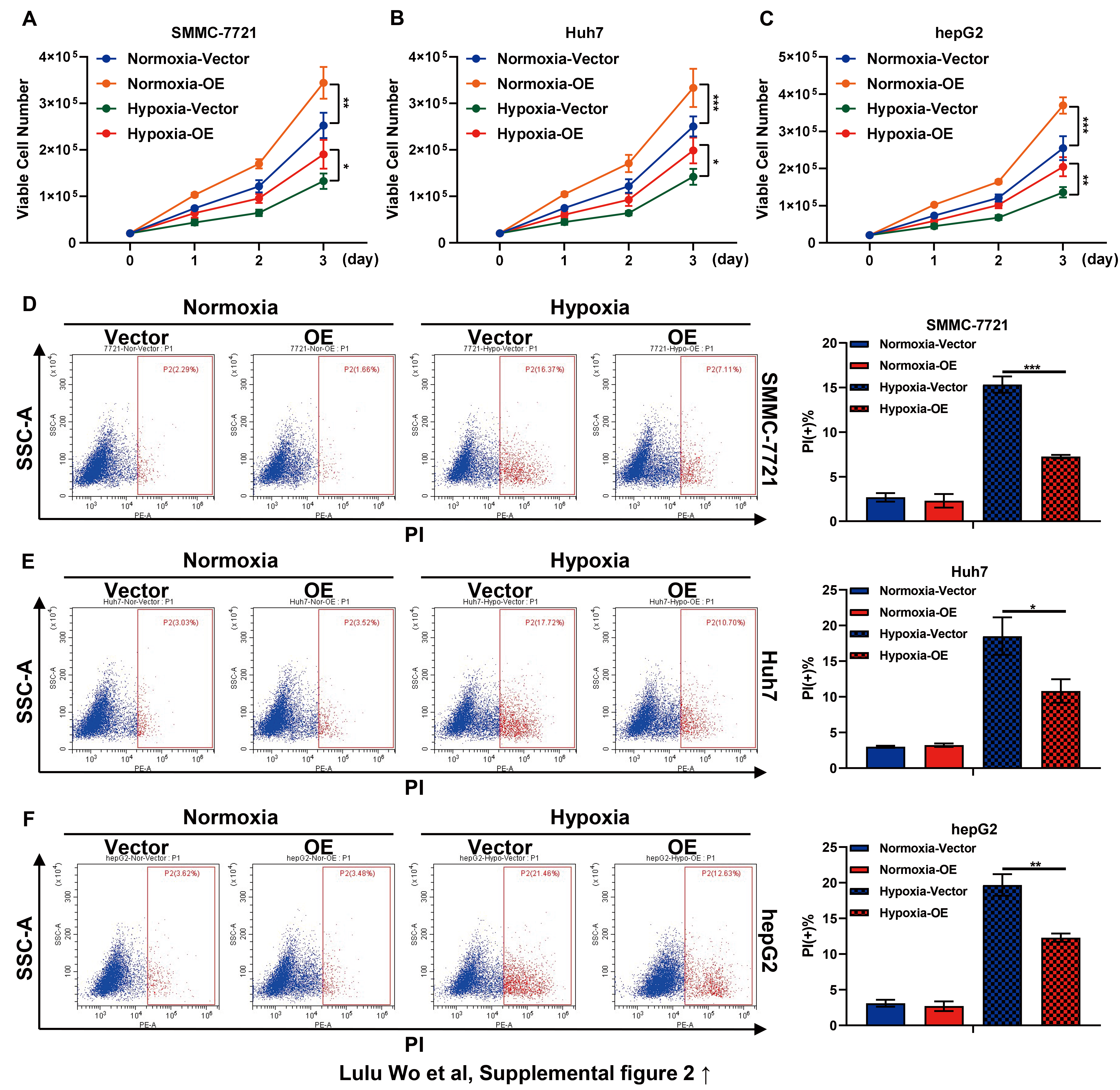

Supplement: Supplementary file 3 — Supplemntal figure 2 [file 41420_2022_917_MOESM3_ESM.png]

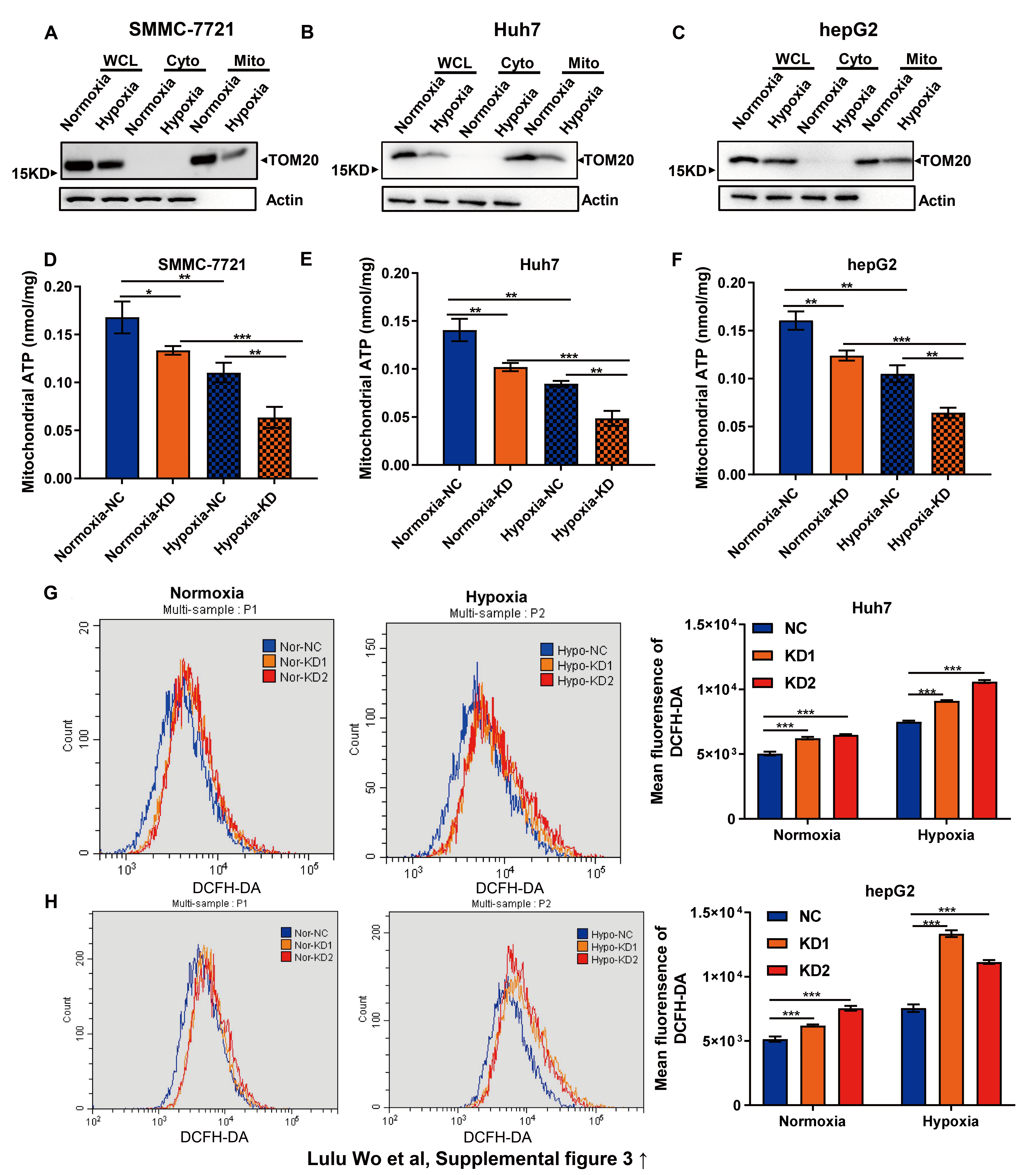

Supplement: Supplementary file 4 — Supplemntal figure 3 [file 41420_2022_917_MOESM4_ESM.png]

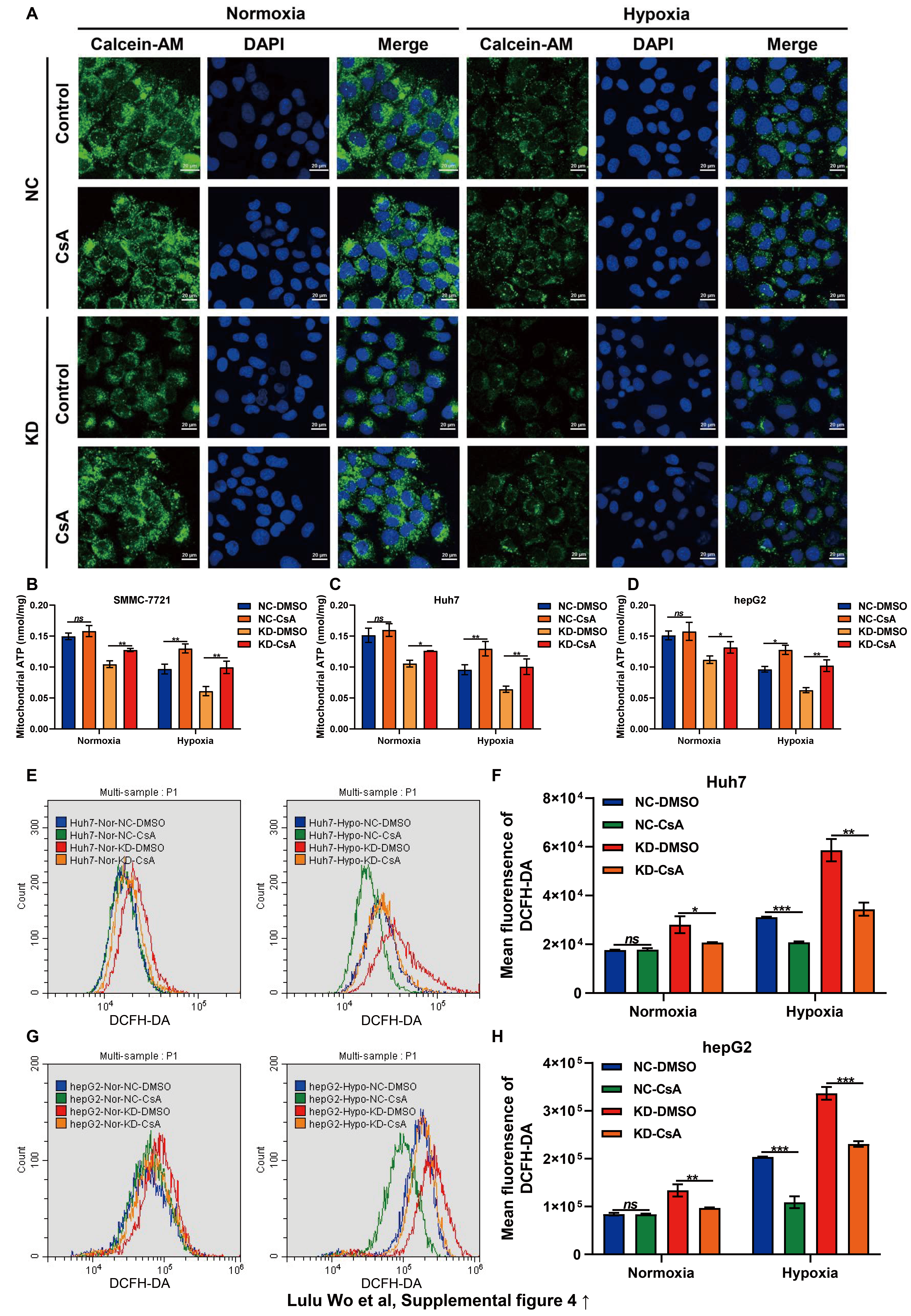

Supplement: Supplementary file 5 — Supplemntal figure 4 [file 41420_2022_917_MOESM5_ESM.png]

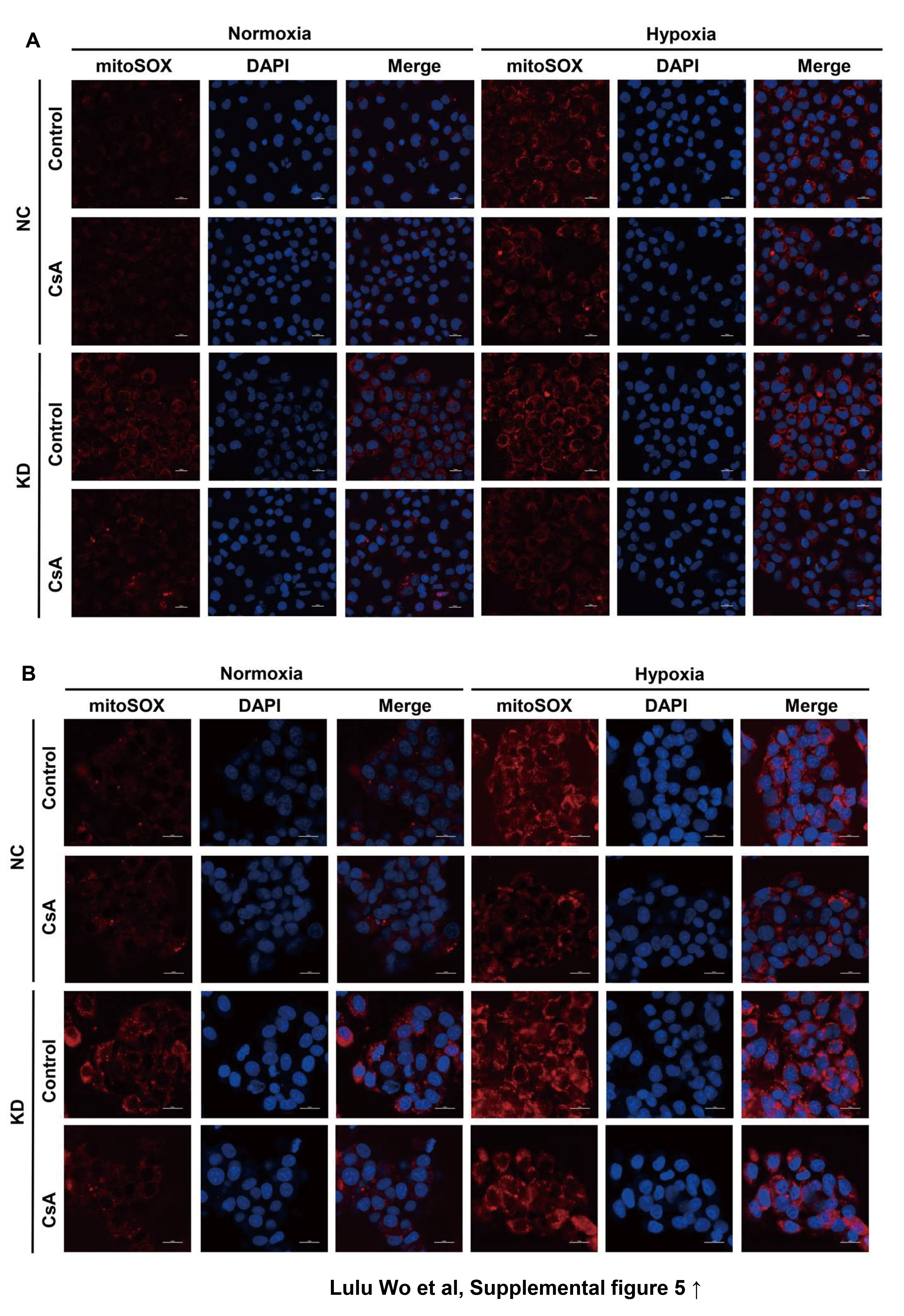

Supplement: Supplementary file 6 — Supplemntal figure 5 [file 41420_2022_917_MOESM6_ESM.png]

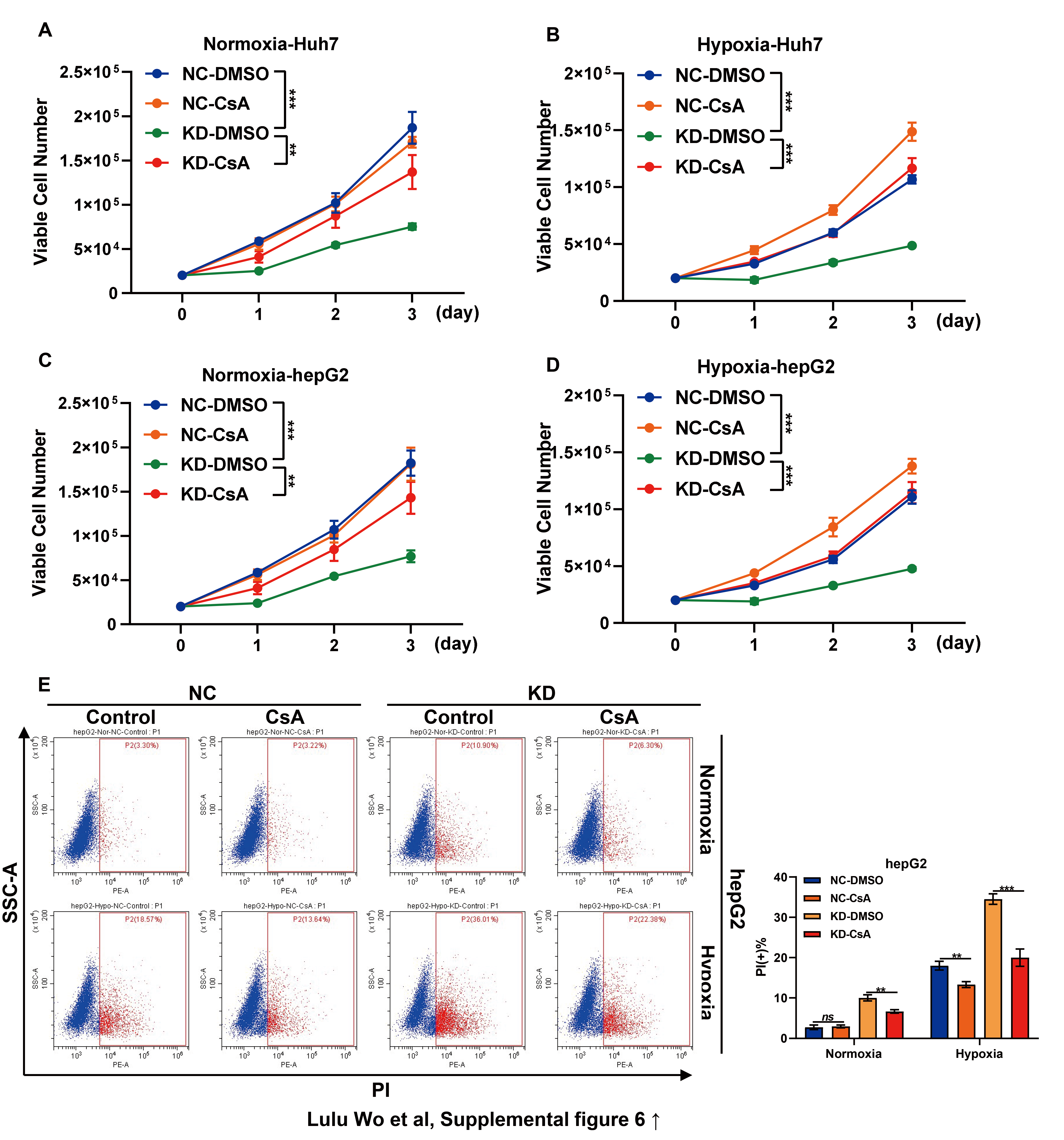

Supplement: Supplementary file 7 — Supplemntal figure 6 [file 41420_2022_917_MOESM7_ESM.png]

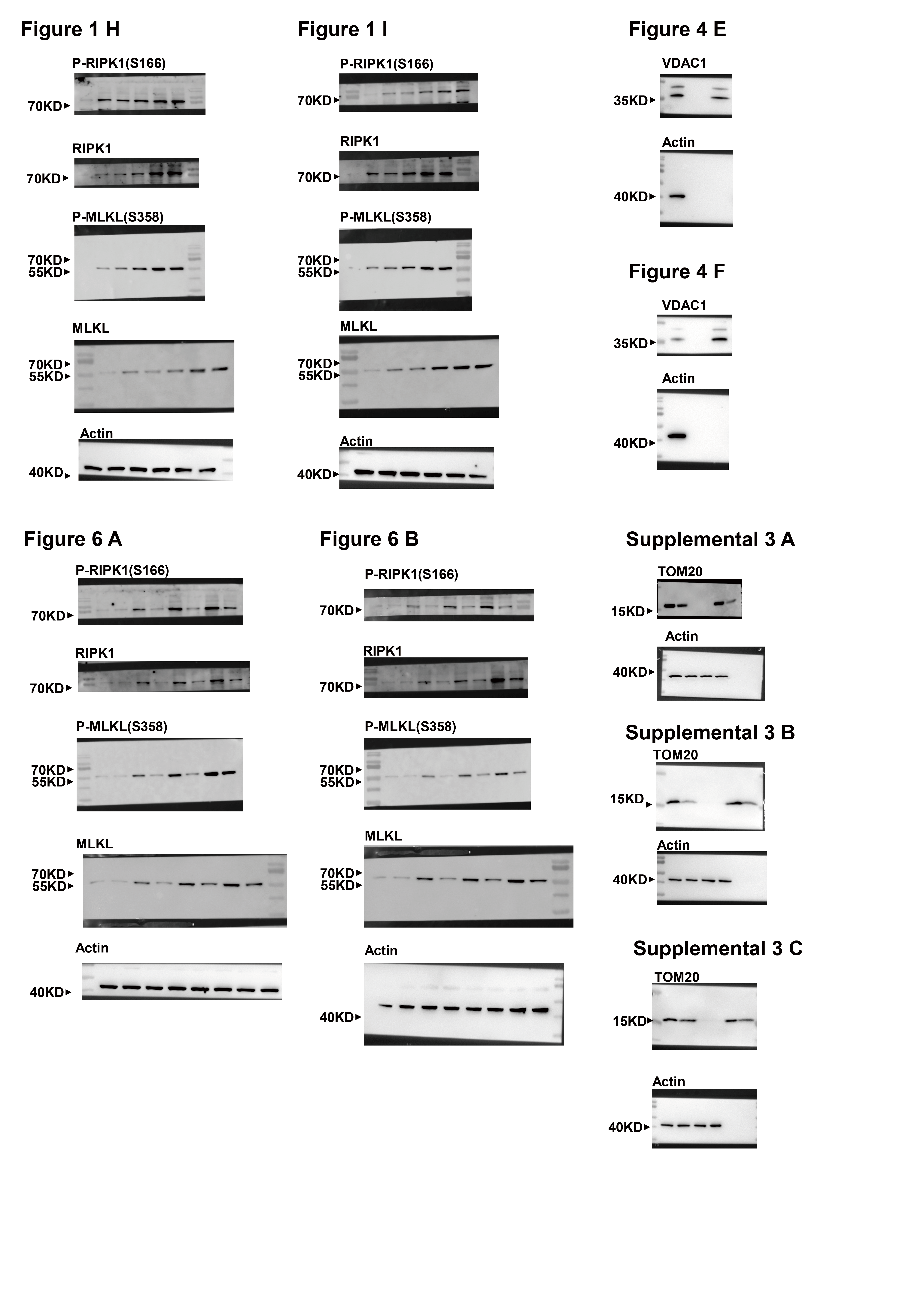

Supplement: Supplementary file 8 — uncropped western blots [file 41420_2022_917_MOESM8_ESM.png]
